# Supplementary material for: Osteogenic Induction with Silicon Hydroxyapatite Using Modified Autologous Adipose Tissue-Derived Stromal Vascular Fraction: In Vitro and Qualitative Histomorphometric Analysis
Source: Materials (Basel). 2022 Feb 28;15(5):1826. doi: 10.3390/ma15051826 (PMC8911855; doi:10.3390/ma15051826)
Supplement: Supplementary file 1 [file materials-15-01826-s001.zip › materials-1575274-supplementary.pdf]

materials

MDPI

Supplementary Materials:

Osteogenic Induction with Silicon Hydroxyapatite Using Modified Autologous Adipose Tissue-Derived Stromal Vascular Fraction: In Vitro and Qualitative Histomorphometric Analysis

Muhammad Marghoob Khan [1], Shadab Ahmed Butt [1], Aqif Anwar Chaudhry [2,*], Amir Rashid [1], Kashif Ijaz [2], Asifa Majeed [1] and Hashmat Gul [1]

Table S1. Blood complete picture of rabbits of group Group I, before and 6weeks after implantation of Si-HA.

S. No	Specimen No	Rabbit No 1 0 days	After 06 weeks	Rabbit No 2 0 days	After 06 weeks	Rabbit No 3 0 days	After 06 weeks	Units
1.	RBC count	4.97	5.90	3.92	5.10	4.39	4.16	m/µL
2.	Hemoglobin	11.5	13.6	12.2	13.1	9.9	12.4	g/µL
3.	Hematocrit	29.5	38.0	31.4	36.0	26.3	37.8	%
4.	MCV	59.4	64.3	58.2	66.3	59.8	81.0	fL
5.	MCH	23.2	22.1	25.1	27.0	22.6	24.6	Pg
6.	MCHC	39	35.9	38.3	33.4	37.8	34.5	g/dl
7.	Platelets	253,000	337,000	201,000	301,000	359,000	259,000	/µL

S. No	Specimen No	Rabbit No 4 0 days	After 06 weeks	Rabbit No 5 0 days	After 06 weeks
1.	RBC count	6.57	3.92	6.02	3.45
2.	Hemoglobin	15.2	11.4	14.3	11.9
3.	Hematocrit	39.9	32.5	37.2	32.4
4.	MCV	60.7	57.0	61.0	57.9
5.	MCH	23.2	29.2	24.1	24.9
6.	MCHC	38.2	32.1	38.9	33.8
7.	Platelets	308,000	279,000	286,000	254,000

Table S2. Blood complete picture of rabbits of group Group II, before and 6weeks after implantation of Si-HA.

S. No	Specimen No	Rabbit No 1 0 days	After 06 weeks	Rabbit No 2 0 days	After 06 weeks	Rabbit No 3 0 days	After 06 weeks	Units
1.	RBC count	5.74	5.12	5.04	5.0	5.08	3.94	m/μL
2.	Hemoglobin	12.6	11.7	11.8	12.3	12.9	12.9	g/μL
3.	Hematocrit	33.1	36.1	30.5	36.9	41.3	34.1	%
4.	MCV	57.7	70.6	60.4	72.4	65.8	71.4	fL
5.	MCH	22.0	22.9	23.4	22.0	20.5	24.7	Pg
6.	MCHC	38.1	32.5	38.7	31.3	31.2	30.7	g/dl
7.	Platelets	370,000	104,000	215,000	333,000	248,000	257,000	/μL

S. No	Specimen No	Rabbit No 4 0 days	After 06 weeks	Rabbit No 5 0 days	After 06 weeks
1.	RBC count	5.5	5.12	5.81	4.72
2.	Hemoglobin	11.8	10.9	12.2	10.3
3.	Hematocrit	38.5	36.1	39.3	36.8
4.	MCV	69.3	70.4	66.7	75.8
5.	MCH	21.3	22.7	20.7	22.0
6.	MCHC	30.7	32.7	31.0	32.7
7.	Platelets	247,000	324,000	399,000	309,000

Table S3. Blood complete picture of rabbits of group Group III, before and 6weeks after implantation of Si-HA.

S. No	Specimen No	Rabbit No 1 0 days	After 06 weeks	Rabbit No 2 0 days	After 06 weeks	Rabbit No 3 0 days	After 06 weeks	Units
1.	RBC count	05	4.2	4	4.2	4.2	4.1	m/μL
2.	Hemoglobin	15	12	16	14.8	10.8	10.3	g/μL
3.	Hematocrit	47.5	36	45	36.2	30.4	28.9	%
4.	MCV	90	82.5	95	82.9	72.3	60.3	fL
5.	MCH	27	26.8	29	26.8	28.5	25.9	Pg
6.	MCHC	33	35.2	34	34.4	33.99	31.9	g/dl
7.	Platelets	25,0000	320000	255,000	30,0000	197,000	167,000	/μL

S. No	Specimen No	Rabbit No 4 0 days	After 06 weeks	Rabbit No 5 0 days	After 06 weeks
1.	RBC count	4.8	3.7	4.2	3.9
2.	Hemoglobin	11.5	12.5	12.3	12.9
3.	Hematocrit	29.6	26.6	26.9	26.0
4.	MCV	57.5	53.0	47.8	51.7
5.	MCH	23.4	23.9	21.9	22.7
6.	MCHC	29.6	26.4	22.8	22.8
7.	Platelets	184,000	191,000	273,000	231,000

Table S4. Blood complete picture of rabbits of group Group IV, before and 6weeks after implantation of Si-HA.

S. No	Specimen No	Rabbit No 1	After 06 weeks	Rabbit No 2	After 06 weeks	Rabbit No 3	After 06 weeks	Units
		0 days		0 days		0 days		
1.	WBC count	9,000	7800	8500	10,050	5300	5700	/μL
2.	RBC count	5.5	4.2	4.8	4.7	4.1	4.9	m/μL
3.	Hemoglobin	16	15.2	13.8	16	13.4	13.8	g/μL
4.	Hematocrit	47	42.8	47	43.9	37.2	38.8	%
5.	MCV	84	18.8	79.7	92	79.7	77.7	fL
6.	MCH	30	30.2	29.3	27.6	28.7	28.7	Pg
7.	MCHC	31.5	32.5	33.3	33	35.9	37.0	g/dl
8.	Platelets	212,000	224,000	235,000	420,000	101,000	231,000	/μL

S. No	Specimen No	Rabbit No 4	After 06 weeks	Rabbit No 5	After 06 weeks
		0 days		0 days	
1.	WBC count	6900	4900	7800	10800
2.	RBC count	3.4	3.8	3.9	3.9
3.	Hemoglobin	11.9	11.5	11.3	13.3
4.	Hematocrit	34.3	34.7	32.0	32.8
5.	MCV	78.3	80.7	78.0	78.7
6.	MCH	22.3	23.5	22.9	26.5
7.	MCHC	38.0	37.0	38.9	36.3
8.	Platelets	243,000	216,000	273,000	291,000
